# Effects of stress on thymic B cells in C57BL/6J mice: phenotypic characterization and mechanistic insights

**DOI:** 10.3389/fimmu.2026.1963346

**Published:** 2026-10-13

**Authors:** Longda Ma, Weibo Shi, Songjun Wang, Bin Cong

**Affiliations:** 1 Postdoctoral Research Station in Basic Medicine, Hebei Medical University, Shijiazhuang, Hebei, China; 2 Collaborative Innovation Center of Forensic Medical Molecular Identification, Hebei Key Laboratory of Forensic Medicine, Department of Forensic Medicine, Hebei Medical University, Shijiazhuang, Hebei, China

**Keywords:** antigen presentation, B cell activation, central tolerance, stress, thymic B cells

## Abstract

**Introduction:**

As an important cause of immune system dysfunction, stress can damage thymic stromal cells, leading to a persistent imbalance in thymic function. Thymic B cells can present autoantigens, playing a critical role in the establishment of central T cell tolerance. However, current research on the effects of stress on thymic B cells remains limited.

**Methods:**

Publicly available scRNA-seq datasets were analyzed to characterize thymic B cell phenotypes and glucocorticoid responsiveness. A restraint stress mouse model was employed to assess stress-induced changes in thymic B cell abundance and phenotype by flow cytometry and histopathology, and transcriptomic alterations by bulk RNA sequencing of sorted thymic B cells.

**Results:**

Thymic B cells exhibited a distinctive memory B cell signature with constitutively activated immune-related pathways, and robust glucocorticoid responsiveness. Restraint stress significantly reduced thymic B cell numbers, increased the proportion of IgD^+^IgM^+^ B cells, and downregulated the expression of CD80 and CD86 in IgD^+^IgM^+^ B cells. Transcriptomic profiling revealed that stress suppressed cell cycle and G2/M checkpoint pathway, inhibited CD40L- and IFN-λ-responsive gene expression, attenuated Aire-dependent gene expression, and diminished the presentation of autoantigens. Furthermore, the expression levels of CD40 and MHC II in most of the thymic B cell subsets were markedly decreased.

**Discussion:**

This study reveals the detrimental impact of stress on thymic B cell. Collectively, stress impairs thymic B cell proliferation and attenuates critical licensing signals, including the CD40L–CD40 and IFN-λ signaling, thereby leading to a reduction in thymic B cell numbers, compromised activation, and impaired antigen-presenting capacity.

## 1 Introduction

Stress is one of the most common disruptors of homeostasis in the general population. Anxiety disorders and depressive disorders each affect approximately 300 million people worldwide, and chronic psychological or physiological stress has become a major contributor to immune dysfunction (1). Stress activates the hypothalamic-pituitary-adrenal (HPA) axis to promote the release of hormones such as glucocorticoids (GCs), leading to thymic atrophy, disruption of the corticomedullary architecture, and damage to thymic stromal cells, thereby causing persistent thymic dysfunction (2–4). Notably, given that the thymus is the central lymphoid organ responsible for T cell development, maturation, and the establishment of central immune tolerance, impaired thymic function can increase the risk of autoimmune diseases (5, 6).

Thymic B cells, a stromal cell population of hematopoietic origin, primarily reside in the medulla and play an irreplaceable role in establishing central T cell tolerance by eliminating self-reactive T cell clones (7–9). It has been demonstrated that B cells express the glucocorticoid receptor (GR) at all developmental stages, and GCs exert a significant inhibitory effect on the proliferation and differentiation of peripheral B cells (10–12). We therefore hypothesized that thymic B cells are directly regulated by stress, which could alter their phenotype and impair their physiological functions. Given the critical roles of thymic B cells, investigating their regulation by stress is of considerable importance. However, current research has focused predominantly on the roles of thymocytes, thymic epithelial cells (TECs), and dendritic cells (DCs) in thymic injury and repair (13), while it remains unclear how stress modulates the function of thymic B cells.

The number of thymic B cells is comparable to that of other thymic stromal cells, such as TECs and DCs, accounting for approximately 0.1%–0.5% of total thymocytes (7, 14). Compared with conventional B cells that mediate humoral immunity in peripheral lymphoid organs, thymic B cells express high levels of MHC class II (MHCII) and activation molecules such as CD80 and CD86 on their surface (14, 15). Thymic B cells can express Aire, a key transcription factor that regulates the expression of tissue-restricted self-antigens (TRAs) and plays a critical role in the maintenance of self-tolerance (8, 16). Based on the expression of IgD and IgM, thymic B cells can be divided into three subgroups: IgD^+^IgM^+^, IgD^-^IgM^+^, and IgD^-^IgM^-^ cells. Under the regulation of key molecules such as CD40L–CD40 and type III interferon (IFN-λ), thymic B cells are activated and undergo class-switch recombination (CSR), evolving from IgD^+^IgM^+^ to IgD^-^IgM^-^ cells, which is essential for their unique function (8, 17, 18).

In the present study, we investigated the impact of stress on thymic B cells. We analyzed the characteristics and stress sensitivity of thymic B cells using public databases. Subsequently, using a restraint stress mouse model, we examined the effects of stress on the number and subset composition of thymic B cells. Our findings demonstrated that stress exerts a significant inhibitory effect on the development and function of thymic B cells.

## 2 Methods

### 2.1 Mice

6-week-old male C57BL/6J mice were randomly divided into three groups: a control group and two stress groups (3 d and 7 d). Mice in the stress groups were subjected to restraint stress for 6–8 h daily at fixed times as previously described (19), while control mice were allowed to move freely but were concurrently deprived of food and water. At the end of the experimental treatment, thymic tissues were harvested for histopathological analysis and flow cytometry. All mice were obtained from Beijing Vital River Laboratory Animal Center and housed under specific pathogen-free (SPF) conditions at a temperature of 23 ± 2 °C, 50% humidity, and a 12-h light/dark cycle. The mice were allowed to acclimate for approximately 1 week before the start of the experiment. All animal experimental procedures were reviewed and approved by the Institutional Animal Care and Use Committee of Hebei Medical University (approval number: IACUC-Hebmu-P2022305).

### 2.2 Histological analysis

After the experimental treatment, the mice were euthanized by cervical dislocation. The thymus tissues from the control, stress 3 d, and stress 7 d groups were collected and fixed in 4% paraformaldehyde for 1 week, followed by dehydration, clearing, and paraffin embedding (n = 6). Tissue sections (4 μm thick) were prepared for subsequent staining. For H&E staining, the sections were deparaffinized with xylene, hydrated through a graded ethanol series, stained with hematoxylin and eosin, and mounted with neutral resin. For immunohistochemical (IHC) staining, after deparaffinization and hydration, the sections were subjected to antigen retrieval by microwave heating in citrate buffer and were treated with hydrogen peroxide to quench endogenous peroxidase activity. Non-specific binding was blocked with 5% BSA (Beyotime) at room temperature for 1 h, followed by incubation with anti-CD19 antibody at 4 °C overnight. The next day, the sections were incubated with HRP-labeled secondary antibody at room temperature for 1 h. Finally, the sections were stained with DAB (Beyotime), counterstained with hematoxylin, mounted with neutral resin. Images were captured using a digital slide scanner (Leica). Relevant antibodies used are shown in the **Supplementary Table S1**.

### 2.3 Flow cytometry

Thymus tissues from mice in the control, stress 3 d, and stress 7 d groups were carefully isolated and gently pressed through a 70 μm cell strainer (Absin) to obtain a single-cell suspension (n = 6). Following centrifugation at 300 × g for 5 min at 4 °C, the cell pellet was resuspended in red blood cell lysis buffer (Absin) and incubated at room temperature for 2 min. Staining buffer (Absin) was then added to stop the lysis reaction. After centrifugation and resuspension, the cells were manually counted using a hemocytometer. Approximately 10^6^ cells were subjected to two staining protocols after incubating with an anti-CD16/32 antibody for 5 min at room temperature to block non-specific binding. For protocol 1, to detect the proportion of B cells, cells were stained with CD19. For protocol 2, due to the low proportion of B cells, cells were subjected to negative selection to deplete CD4^+^ and CD8α^+^ T cells (as described in Section 2.4) prior to staining with CD19, CD40, CD80, CD86, and MHC II. Cells were incubated with the corresponding antibodies in the dark at 4 °C for 20 min. Following washing, the cells were incubated with 7-aminoactinomycin D (7-AAD, Beyotime) in the dark at 4 °C for 5 min (1:50), and then analyzed on a CytoFLEX LX flow cytometer (Beckman Coulter). The blank tubes and single-stained tubes for each fluorescence channel were processed identically to the formal samples to establish voltage and compensation parameters. All flow cytometry data were uniformly analyzed using FlowJo (v10.8.1). Specifically, FSC-A vs. SSC-A was used to exclude debris, FSC-A vs. FSC-H to exclude doublets, and 7-AAD vs. SSC-A to gate live cells. The detailed gating strategies are illustrated in **Supplementary Figure S5A** (for protocol 1) and **Supplementary Figure S5B** (for protocol 2). Following cell gating, the median fluorescence intensity (MFI) of CD40, CD80, CD86, and MHC-II was calculated for each cell population using the function “Add Statistic”. Relevant antibodies used are shown in the **Supplementary Table S1**.

### 2.4 Isolation of B cell

Thymic single-cell suspensions were prepared and counted as described above. Briefly, 10^7^ cells were incubated with biotin-conjugated anti-CD4 and anti-CD8α antibodies at 4 °C for 30 min. After washing, the cells were incubated with streptavidin-conjugated magnetic beads (BD Biosciences) at 4 °C for 30 min, thereby allowing CD4^+^ or CD8α^+^ cells to be re-labeled with the magnetic beads. The tube containing the sample was then placed in a cell separation magnet (BD Biosciences) and left undisturbed for 10 min, allowing the magnetic bead-bound cells to migrate toward and attach to the tube wall under the magnetic field. The suspension, which was free of CD4^+^ or CD8α^+^ cells, was carefully transferred to a new flow cytometry tube. This magnetic separation procedure was repeated twice to ensure adequate depletion of CD4^+^ or CD8α^+^ cells. These cells were subsequently subjected to flow cytometry Protocol 2 (Section 2.3) or utilized for FACS sorting. For sorting, cells were blocked with an anti-CD16/32 antibody, then incubated with CD19 (20 min) and stained with 7-AAD (5 min) at 4 °C in the dark. Finally, CD19^+^ B cells were sorted using a CytoFLEX SRT cell sorter (Beckman Coulter) and collected in PBS supplemented with 50% FBS. The sorted cells were re-stained with CD19 and 7-AAD and analyzed by flow cytometry. Samples with >95% purity and >90% viability were subjected to transcriptome sequencing. Relevant antibodies used are shown in the **Supplementary Table S1**.

### 2.5 Bulk RNA-seq and data processing

Total RNA was extracted from sorted cells of the control, stress 3 d, and stress 7 d groups using the RNeasy Micro Kit (QIAGEN) according to the manufacturer’s instructions (n = 3), and reverse transcribed into cDNA using the QuantiTect Reverse Transcription Kit (QIAGEN). cDNA quality was assessed using a Qubit 4.0 fluorometer and an Agilent 4200 Bioanalyzer. Sequencing libraries were prepared using the TruePrep DNA Library Prep Kit V2 for Illumina (Vazyme) and sequenced on the NovaSeq X Plus (Illumina) platform. Reads were aligned to the reference genome (mm39) using HISAT2 (20), and gene-level read counts were generated with featureCounts to obtain the raw count matrix (21). Genes with zero counts in more than 6 samples were filtered out as lowly expressed. Differential expression analysis was performed using DESeq2 (22), and differentially expressed genes (DEGs) were defined as those with |log_2_ (fold change)| (log_2_FC) ≥ 1 and *P* < 0.05. KEGG, GO, and GSEA enrichment analyses were conducted using the clusterProfiler package (23), and pathways with a *P* value < 0.05 were considered statistically significant.

### 2.6 Analysis of public single-cell RNA-sequencing data

Single-cell RNA-sequencing datasets were obtained from the GEO database under accession numbers GSE234188 and GSE244229. For GSE234188, quality control and data analysis were performed using the Seurat package according to the parameters described in the original publications (9, 24, 25). Uniform manifold approximation and projection (UMAP) was applied to visualize the transcriptional differences among B cells from different tissues. DEGs between thymic B cells and B cells from other tissues (spleen, lymph node, bone marrow, and blood) was identified using the FindMarkers function in Seurat with the default Wilcoxon rank-sum test (logfc.threshold = 0, min.pct = 0.1), and filtered by an adjusted P value (*P_adj_*) < 0.05 and |log_2_FC| > 1. For GSE244229, gene expression profiles of B cells were extracted based on the pre-annotated cell classifications (26). DEGs between the 18 h group (after treatment with dexamethasone) and control group was identified using FindMarkers with the Wilcoxon rank-sum test (logfc.threshold = 0, min.pct = 0.05), and filtered by *P_adj_* < 0.05 and |log_2_FC| > 0.25. Enrichment analyses, including KEGG pathway and GO analyses, were performed on the DEGs, with KEGG pathways and GO terms showing *P_adj_* < 0.05 considered significantly enriched. GSEA was performed using all genes, and gene sets with *P* < 0.05 were considered significantly enriched.

### 2.7 Statistical analysis

Statistical analysis was performed using GraphPad Prism (v9.5.0). Normality within each group was assessed using the Shapiro-Wilk test, and homogeneity of variances was assessed using the Brown-Forsythe test. For variables where all groups satisfied the normality assumption, data are presented as mean ± standard deviation (SD); one-way ANOVA followed by Dunnett’s *post hoc* test was used when variances were equal, and Welch’s ANOVA with Dunnett’s T3 *post hoc* test was applied when variances were unequal. For variables where any group failed the normality test, data are presented as median and interquartile range (IQR), and the Kruskal-Wallis test followed by Dunn’s *post hoc* test was used. A *P* value < 0.05 was considered statistically significant. *P* > 0.05, *P* < 0.05, *P* < 0.01, *P* < 0.001, and *P* < 0.0001 were represented by ns, *, **, ***, **** respectively. Additionally, figures were prepared using RStudio (v4.4.1) and Adobe Illustrator 2024 (v.28.0).

## 3 Results

### 3.1 Public single-cell data analysis reveals that thymic B cells are potential targets of stress

Although the effects of stress on peripheral B cells are well documented, its impact on thymic B cells remains largely unexplored (10–12). Thymic B cells have been reported to exhibit distinct phenotypes compared to peripheral B cells (14, 15). To further elucidate these differences, we analyzed their phenotypic characteristics using the GSE234188 dataset, which contains single-cell sequencing data of CD19^+^ B cells from multiple mouse tissues, including bone marrow, blood, spleen, lymph nodes, and thymus (9). UMAP visualization revealed significant differences in the composition of clusters of thymic B cells compared with B cells in other tissues, such as blood and lymph nodes (**Supplementary Figure S1A**). *Aire*, a key regulator of TRAs expression, was predominantly expressed in thymic B cells rather than in B cells from other tissues (**Supplementary Figure S1B**). Differential expression analysis identified 183 DEGs in thymic B cells (106 upregulated, 77 downregulated) relative to other B cells (**Supplementary Figure S1C**). Enrichment analysis of the upregulated DEGs highlighted significant involvement of pathways such as measles, Epstein-Barr virus infection, and antigen processing and presentation (**Figure 1A**). Similarly, GO analysis revealed an enrichment for terms associated with viral response and defense (**Supplementary Figure S1D**). For the downregulated DEGs, no significantly enriched KEGG pathways were identified; only 40 GO terms were enriched, and the top 10 most significant BP terms were listed in **Supplementary Table S2**. GSEA indicated that the signature genes of memory B cells were significantly upregulated in thymic B cells (27), whereas those of naive B cells were downregulated (**Figures 1B, C**). Furthermore, thymic B cells broadly expressed the GR gene *Nr3c1* (**Figure 1D**) and the adrenergic receptor gene *Adrb2* (**Figure 1F**), whereas a minor subset additionally expressed the mineralocorticoid receptor gene *Nr3c2* (**Figure 1E**).

**Figure 1:**
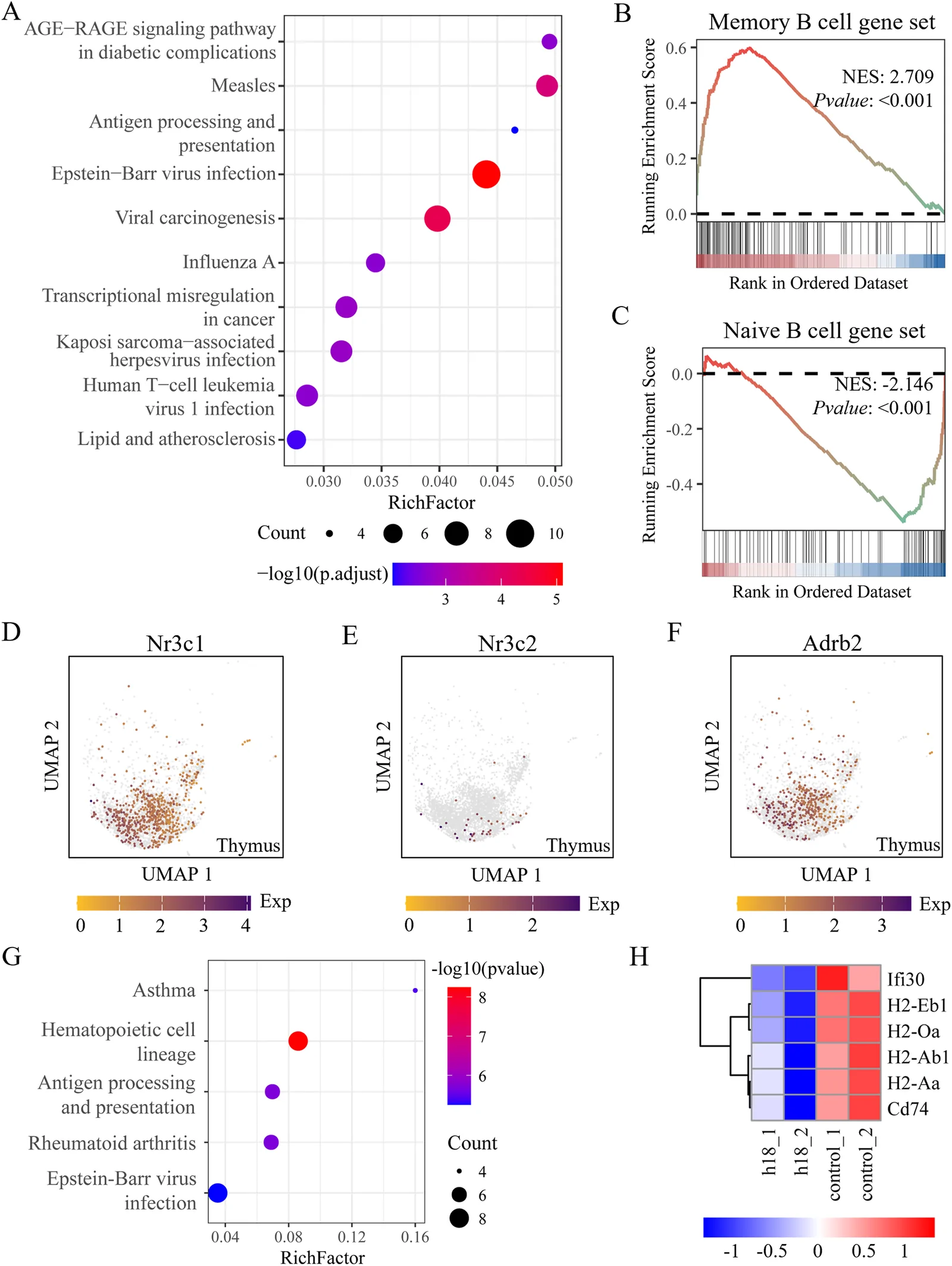
Characterization of thymic B cells and their responsiveness to GCs stimulation using public single-cell datasets. **(A-F)** Analysis of the single-cell dataset GSE234188; **(G, H)** Analysis of B cell transcriptomes extracted from the single-cell dataset GSE244229. **(A)** KEGG pathway enrichment analysis of DEGs between thymic and non-thymic B cells (*P_adj_* < 0.05; top 10 most significant pathways shown). **(B, C)** GSEA of thymic B cells versus B cells from other tissues using signature gene sets for memory B cell **(B)** or a naive B cell signature **(C)**. Genes were ranked by fold change in descending order. **(D–F)** Feature plots showing *Nr3c1* **(D)**, *Nr3c2* **(E)**, and *Adrb2* **(F)** expression in thymic B cells; gray indicates undetectable expression. **(G)** KEGG pathway enrichment analysis of DEGs in the 18 h group relative to the control (*P_adj_* < 0.05; top 5 most significant pathways shown). **(H)** Heatmap of antigen presentation related DEGs in the 18 h group relative to the control.

Although phenotypically distinct from peripheral B cells, the high expression of stress hormone receptors in thymic B cells indicates they are also targets of stress. Accordingly, we analyzed their transcriptomic response to GCs using the GSE244229 dataset, which profiles mouse thymic stromal cells treated with GCs (18 h or 4 d) or PBS (control). From the extracted B cell data, 83 DEGs were identified in the 18 h group relative to controls (33 upregulated, 50 downregulated; **Supplementary Figure S1E**). KEGG pathway analysis highlighted pathways associated with antigen processing and presentation and hematopoietic cell lineage (**Figure 1G**). Consistently, GO analysis identified terms related to MHC II-mediated peptide antigen presentation and B cell activation (**Supplementary Figure S1F**). Notably, MHC II–related genes within this pathway, such as *H2-Eb1*, *H2-Aa*, and *H2-Ab1*, were downregulated in the 18 h group (**Figure 1H**). Furthermore, GSEA demonstrated a significant upregulation of naive B cell signature genes (**Supplementary Figure S1G**) and downregulation of cell cycle-associated genes in the 18 h group (**Supplementary Figure S1H**) (27).

### 3.2 Stress suppresses thymic B cell proliferation and reduces their abundance

Analysis of public datasets supports the hypothesis that stress may regulate thymic B cells. To further investigate this regulatory effect, we employed a classic restraint stress model to further investigate the effects of stress on thymic B cells (19, 28). 6-week-old male C57BL/6J mice were assigned to three groups based on restraint duration: control, stress 3 d, and stress 7 d (**Figure 2A**). Stress exposure induced marked thymic atrophy in stress groups (**Figure 2B**). Specifically, thymus weight decreased from 55.45 ± 5.33 mg in controls to 23.52 ± 7.79 mg and 16.32 ± 5.07 mg in the stress 3 d and 7 d groups, respectively (**Supplementary Figure S2A**). Total thymocyte counts declined by 93.78% and 97.43% following 3 and 7 days of stress (**Supplementary Figure S2B**). Histopathological examination revealed substantially reduced cortical cellularity with blurred corticomedullary boundaries (**Supplementary Figure S2C**). IHC staining for CD19 showed that thymic B cells were predominantly localized in the medulla (**Figure 2C**). Although their distribution pattern remained largely unchanged under stress, the relative proportion of thymic B cells appeared to increase (**Figure 2C**); spleen tissue was used as a positive control (**Supplementary Figure S6**). Flow cytometry confirmed a significant increase in the frequency of thymic B cells among total thymocytes, from 0.32% ± 0.02% in controls to 1.63% ± 0.42% (stress 3 d) and 2.22% ± 0.64% (stress 7 d) (**Figure 2D**). Nevertheless, the absolute number of thymic B cells decreased by 70.48% and 83.31% in the stress 3 d and 7 d groups, respectively (**Figure 2E**).

**Figure 2:**
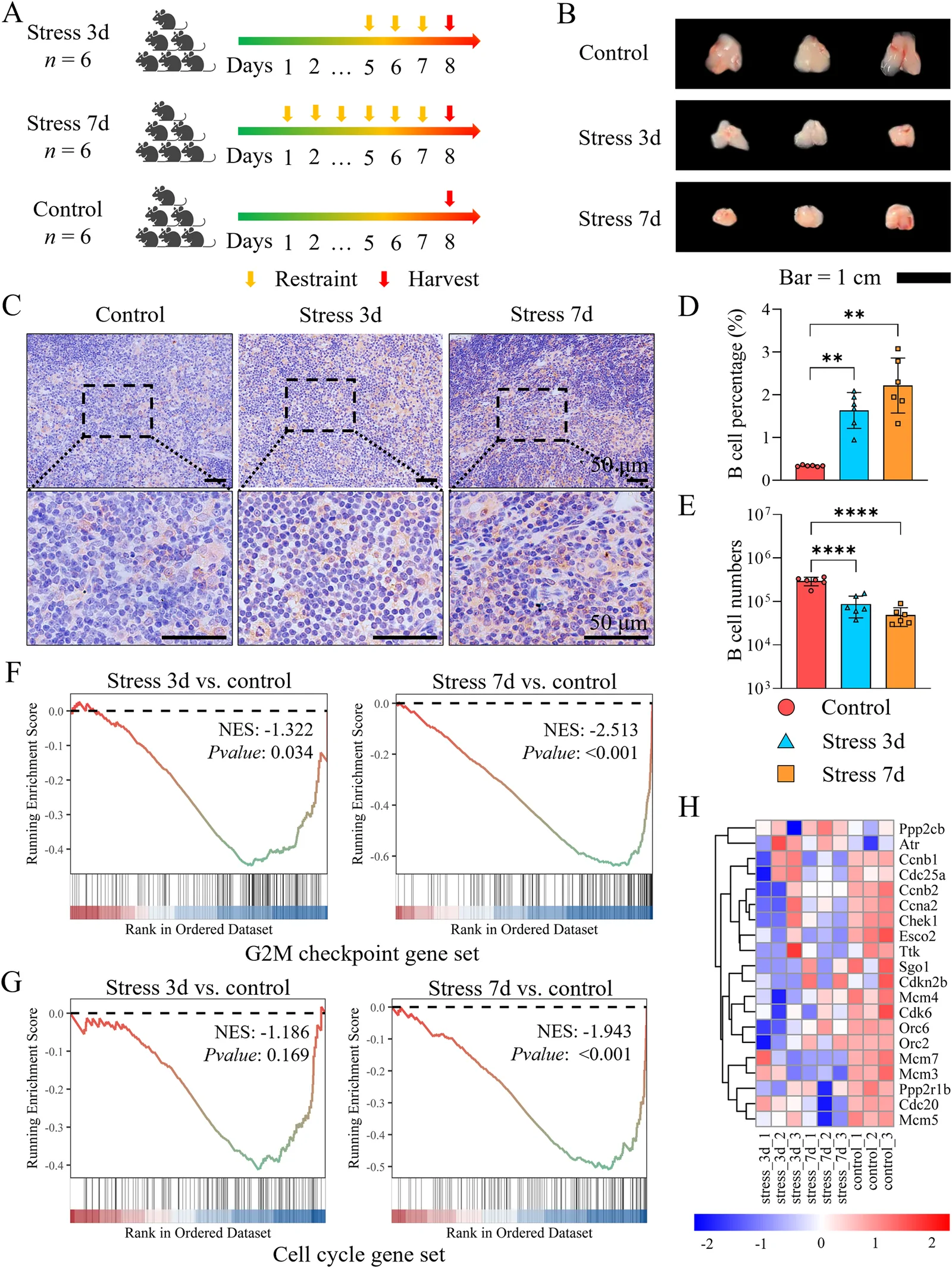
Effects of stress on thymic B cell abundance and proliferation-related pathways in mice. **(A)** Schematic illustration of the experimental design. **(B)** Representative gross images of thymus in control and stressed mice (n = 3). **(C)** IHC staining of B cells with CD19 in control and stressed mice (Bar = 50 μm, n = 3). **(D, E)** Flow cytometry quantification of the proportion **(D)** and absolute number **(E)** of CD19^+^ thymic B cells across the three groups (n = 6). **(F, G)** GSEA of the stress 3 d or stress 7 d group versus the control using G2/M checkpoint gene set **(F)** or cell cycle gene set **(G)**. Transcriptome data were obtained from CD19^+^ B cells sorted from the thymus of mice (n = 3). **(H)** Heatmap of cell cycle related DEGs in the stress 3 d and stress 7 d groups relative to the control. DEGs were defined as |log_2_FC| ≥ 1 and *P* < 0.05 (n = 3). Data are presented as mean ± SD. Statistical significance was determined by one-way ANOVA followed by Dunnett’s *post hoc* test, or Welch’s ANOVA followed by Dunnett’s T3 *post hoc* test **(D)**. **, *P* < 0.01; ****, *P* < 0.0001.

To elucidate the underlying mechanisms driving the reduction of thymic B cells, we isolated CD19^+^ B cells from the thymus and performed RNA sequencing. Differential expression analysis identified 843 DEGs in the stress 3 d group (262 upregulated, 581 downregulated; **Supplementary Figure S2D**) and 308 DEGs in the stress 7 d group (136 upregulated, 172 downregulated; **Supplementary Figure S2E**). GSEA indicated a significant suppression of cell proliferation. Specifically, signature genes associated with the G2M checkpoint were significantly downregulated in both the stress 3 d group (NES = -1.322, *P* = 0.034) and the stress 7 d group (NES = -2.513, *P* < 0.001), while general cell cycle signature genes were also suppressed in the stress 7 d group (NES = -1.943, *P* < 0.001). Consistently, key cell cycle regulators, including *Cdc20*, *Mcm3*, *Mcm7*, and *Ccnb1*, were significantly downregulated in the stress groups compared to controls (**Figure 2H**).

### 3.3 Stress inhibits the activation of thymic B cell and expands the IgD^+^IgM^+^ subset

Thymic B cells can be classified into three subsets based on IgD and IgM expression (**Figure 3A**). In control mice, the IgD^+^IgM^+^ subset was predominant (48.23% ± 5.64%), followed by IgD^-^IgM^+^ (28.30% ± 4.15%) and IgD^-^IgM^-^ subsets (20.37% ± 2.46%). Stress exposure induced a significant composition change: the proportion of IgD^+^IgM^+^ cells expanded substantially, reaching 61.97% ± 3.50% (stress 3 d) and 57.27% ± 4.29% (stress 7 d), whereas IgD^-^IgM^+^ and IgD^-^IgM^-^ populations declined under stress conditions (**Figure 3A**). The accumulation of IgD^+^IgM^+^ cells, which has not undergone CSR, suggested that stress might impede the activation of thymic B cells. We assessed the expression of classical activation markers to test this hypothesis. Overall, surface CD80 levels dropped by 42.71% in the stress 3 d group, while CD86 levels transiently increased by 30.60% before normalizing by day 7 (**Supplementary Figures S3A, B**). Subset-specific analysis revealed that in IgD^+^IgM^+^ cells, CD80 was markedly decreased (**Figure 3B**), whereas CD86 initially rose but subsequently declined (**Figure 3C**). Similar trends were observed in the IgD^-^IgM^+^ subset (**Supplementary Figures S3C, D**). In contrast, the IgD^-^IgM^-^ subset, which has undergone CSR, exhibited significant upregulation of both CD80 and CD86 (**Supplementary Figures S3E, F**).

**Figure 3:**
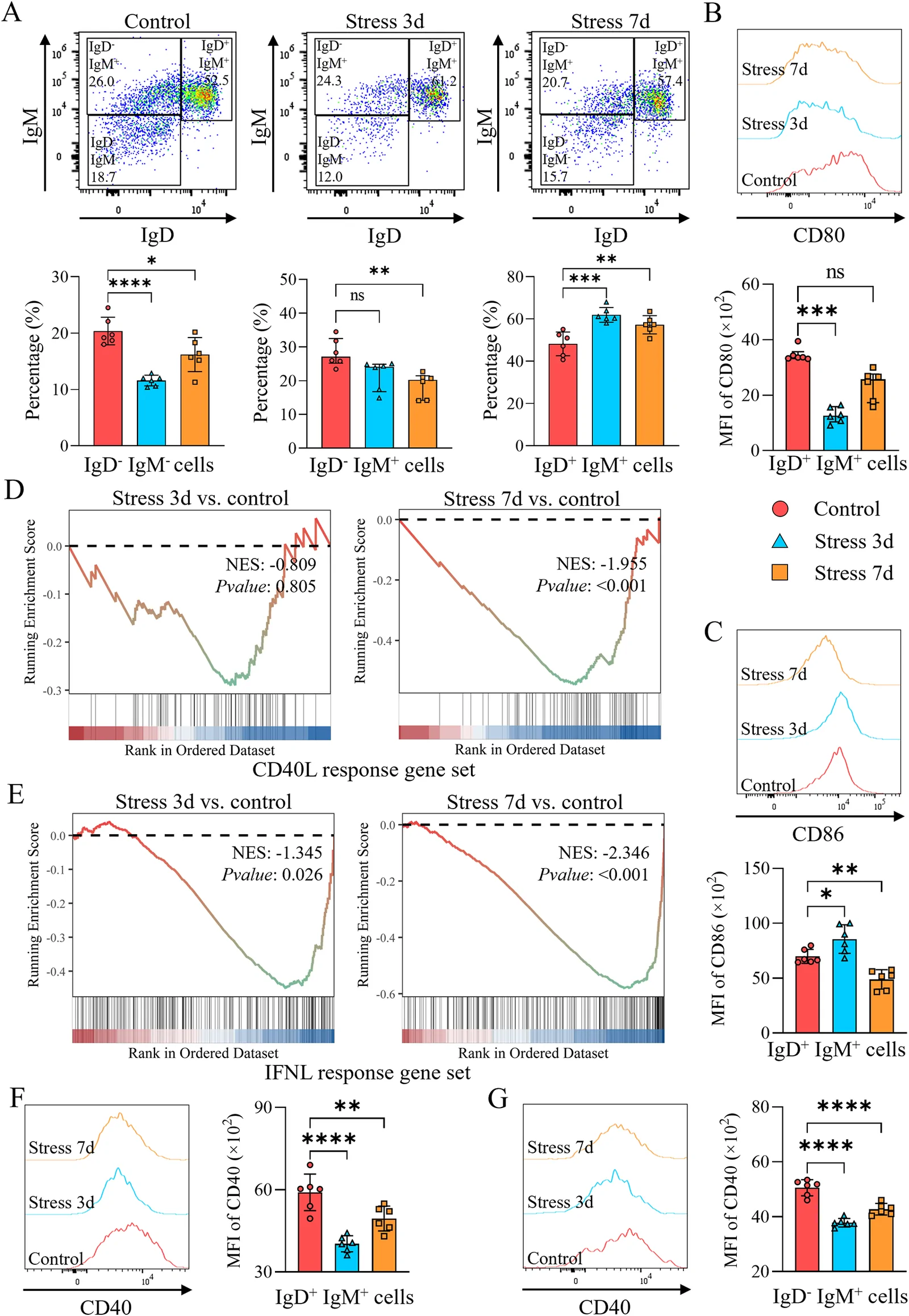
Stress suppresses thymic B cell activation and alters subset composition. **(A)** Proportions of IgD^+^IgM^+^, IgD^-^IgM^+^, and IgD^-^IgM^-^ B cells among CD19^+^ B cells in control and stressed mice (n = 6). **(B, C)** Flow cytometry analysis of CD80 **(B)** and CD86 **(C)** expression in IgD^+^IgM^+^ thymic B cells from control and stressed mice (n = 6). **(D, E)** GSEA of the stress 3 d or stress 7 d group versus the control using a CD40L response gene set **(D)** or an IFN-λ response gene set **(E)**. Transcriptome data were obtained from CD19^+^ B cells sorted from the thymus of mice (n = 3). **(F, G)** CD40 expression in thymic B cell subsets from control and stressed mice, gated on IgD^+^IgM^+^ **(F)** or IgD^-^IgM^+^ **(G)** cells (n = 6). Representative flow cytometry dot plots or histograms for each group are shown in **(A–C, F, G)**. Data are presented as mean ± SD or median (IQR). Statistical significance was determined by one-way ANOVA followed by Dunnett’s *post hoc* test, or Kruskal-Wallis test followed by Dunn’s *post hoc* test **(A)**, middle panel; **(B)**. ns, *P* > 0.05; *P < 0.05; **P < 0.01; ***P < 0.001; ****P < 0.0001.

We next investigated signaling pathways governing B cell activation. GSEA revealed significant downregulation of the IFN-λ response pathway in both stress groups (stress 3 d: NES = -1.345, *P* = 0.026; stress 7 d: NES = -2.346, *P* < 0.001), as well as the CD40L response pathway at day 7 (NES = -1.955, *P* < 0.001) (**Figures 3D, E**) (29, 30). Consistent with the attenuation of CD40L signaling, surface CD40 expression on total thymic B cells decreased by 13.25% at day 3 before recovering to baseline at day 7 (**Supplementary Figure S3G**). This downregulation was particularly pronounced in the IgD^+^IgM^+^ and IgD^-^IgM^+^ subsets. At the subset level, CD40 was significantly decreased in both IgD^+^IgM^+^ (**Figure 3F**) and IgD^-^IgM^+^ (**Figure 3G**) cells. Opposing this trend, the IgD^-^IgM^-^ subset exhibited a marked increase in CD40 expression (**Supplementary Figure S3H**).

### 3.4 Stress impairs the antigen processing and presentation capacity of thymic B cells

To elucidate the mechanism of the regulatory effect of stress on thymic B cells, we performed KEGG and GO enrichment analyses. In the stress 3 d group, enriched KEGG pathways were primarily associated with viral infectious diseases and signal transduction (**Supplementary Figure S4A**), whereas in the stress 7 d group, they predominantly involved cell growth and death (**Supplementary Figure S4B**). GO analysis identified terms associated with B cell-mediated immunity and the production of molecular mediators at day 3 (**Supplementary Figure S4C**), shifting toward mitotic processes such as sister chromatid segregation at day 7 (**Supplementary Figure S4D**).

Given our prior observation that GCs regulate antigen processing and presentation in thymic B cells (**Figure 1G**), we suspected that stress might compromise their ability to establish and maintain central T cell tolerance. Using cell type signature gene sets (MSigDB M8), we assessed the expression of self-antigens by GSEA (9). 3 of 4 significantly altered self-antigen gene sets were downregulated in the stress 3 d group (**Figure 4A**). This suppressive effect was more pronounced at day 7, where 13 of 15 altered sets were downregulated (**Figure 4A**). Notably, the Aire-dependent gene set, essential for TRAs expression in thymic B cells, was significantly suppressed in the stress 7 d group (NES = -1.511, *P* = 0.028; **Figure 4B**) (31).

**Figure 4:**
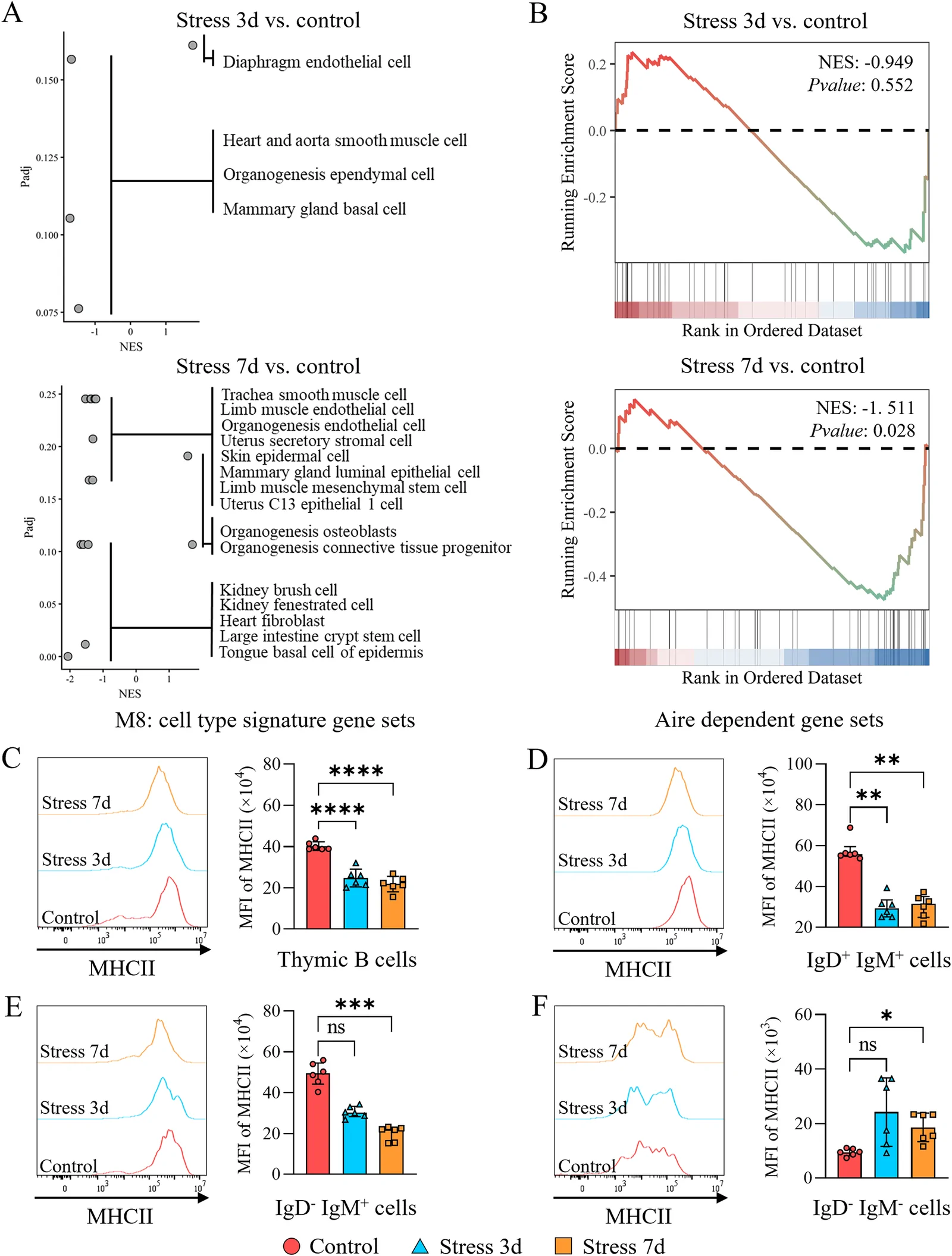
Stress suppresses the antigen processing and presentation capacity of thymic B cells. **(A)** GSEA of the stress 3 d and stress 7 d groups versus the control using cell type signature gene sets (MSigDB, M8). Transcriptome data were obtained from CD19^+^ B cells sorted from the thymus of mice (n = 3). Significance thresholds: *P* < 0.05 and *P_adj_* < 0.25. **(B)** GSEA of the stress 3 d and stress 7 d groups versus the control using Aire dependent gene sets (n = 3). **(C-F)** Flow cytometry analysis of MHC II expression in thymic B cells from control and stressed mice (n = 6), gated on total CD19^+^ cells **(C)**, IgD^+^IgM^+^ cells **(D)**, IgD^-^IgM^+^ cells **(E)**, and IgD^-^IgM^-^ cells **(F)**. Representative histograms are shown in **(C–F)**. Data are presented as mean ± SD or median (IQR). Statistical significance was determined by one-way ANOVA followed by Dunnett’s *post hoc* test, or Welch’s ANOVA followed by Dunnett’s T3 *post hoc* test **(F)**, or Kruskal-Wallis test followed by Dunn’s *post hoc* test **(D, E)**. ns, *P* > 0.05; *P < 0.05; **P < 0.01; ***P < 0.001; ****P < 0.0001.

We further examined MHC II expression on thymic B cells. Stress exposure markedly reduced MHC II levels on total thymic B cells (**Figure 4C**). This downregulation was consistent across the major subsets. MHC II was significantly downregulated in both IgD^+^IgM^+^ (−48.07% at stress 3 d; −47.38% at stress 7 d; **Figure 4D**) and IgD^-^IgM^+^ (−59.21% at stress 7 d; **Figure 4E**) B cells. Intriguingly, the IgD^-^IgM^-^ subset exhibited markedly elevated MHC II expression (**Figure 4F**).

## 4 Discussion

Stress is an important driver of immune dysfunction, and its deleterious effects on thymocytes, TECs, and DCs have been extensively investigated (13). However, whether stress exerts a regulatory influence on thymic B cells, a population of antigen-presenting cells (APCs) essential for the maintenance of central T cell tolerance, remains largely unexplored. Here, we characterized the phenotype and GCs responsiveness of thymic B cells and demonstrated that stress impairs their proliferation, activation, and antigen-presenting function.

Thymic B cells are functionally distinct from B cells in other tissues. By presenting autoantigens and participating in the negative selection of CD4 single-positive (SP) thymocytes, thymic B cells play a non-redundant role in establishing and maintaining central T cell tolerance (7–9). This functional specialization is supported by a distinctive phenotype. Thymic B cells exhibited a higher proportion of IgD^-^IgM^-^ cells and elevated expression of MHC II, CD80, and CD86 (14, 15). Transcriptomically, thymic B cells formed a discrete cluster separated from B cells in other tissues and displayed a gene signature characteristic of memory B cells (**Figure 1B**; **Supplementary Figure S1A**). KEGG and GO enrichment analyses revealed constitutive activation of immune-related pathways in thymic B cells even in the absence of exogenous antigen stimulation (**Figure 1A**; **Supplementary Figure S1D**). We next investigated the responsiveness of thymic B cells to stress. Mechanistically, stress activates the HPA axis, triggering the release of GCs and adrenaline (2). The broad expression of receptors *Nr3c1* and *Adrb2* on thymic B cells provides a molecular basis for direct stress-mediated regulation of these cells (**Figures 1D, F**). Consistently, analysis of the GSE244229 single-cell dataset indicated that stress impairs thymic B cell functions, including antigen presentation and proliferation (**Figures 1G, H**; **Supplementary Figure S1H**).

Thymic B cells reside predominantly in the thymic medulla and account for approximately 0.1–0.5% of total thymic cells in mice (7, 14, 32). The thymic B cell pool is maintained by two principal sources: *in situ* differentiation of precursor cells and recruitment of circulating B cells. The thymus contains pre-B and pro-B cells that actively proliferate and can develop into naive B cells independently of the bone marrow microenvironment (7). Circulating B cells that enter the thymus can also acquire thymic B cell phenotypic features (8, 15). Our data demonstrate that stress induces a marked reduction in thymic B cell numbers, which may be attributable to multiple mechanisms. GCs, the key stress hormones, not only induces thymic atrophy but can also directly trigger B cell apoptosis (33–35). Concurrently, stress significantly downregulates genes associated with the G2M checkpoint and cell cycle pathways (**Figures 2F–H**), indicating suppression of thymic B cell proliferation. Furthermore, under non-inflammatory conditions, the homing of peripheral B cells to the thymus is insufficient to replenish the thymic B cell pool (36).

Thymic B cells undergo a specialized activation process termed “licensing”, during which they undergo CSR, transitioning from an IgD^+^IgM^+^ to an IgD^-^IgM^-^ phenotype (8, 17, 18). While the precise mechanism underlying thymic B cell licensing remains incompletely defined, current evidence suggests dependency on the unique thymic microenvironment. Specifically, self-reactive CD4^+^ SP thymocytes engage with thymic B cells to provide essential CD40L signals (8), while medullary thymic epithelial cells (mTECs) secrete IFN-λ, delivering critical cytokine signals for activation (18). Accordingly, knockdown of either the IFN-λ receptor or CD40L profoundly impairs CSR in thymic B cells (8, 18). Our results demonstrated that stress inhibits “licensing” process, as evidenced by a decreased proportion of IgD^-^IgM^-^ cells with an increase in IgD^+^IgM^+^ cells (**Figure 3A**), as well as markedly reduced expression of CD80 and CD86 on IgD^+^IgM^+^ cells (**Figures 3B, C**). Further studies revealed that stress significantly downregulates signature genes associated with both IFN-λ and the CD40L response pathway (**Figures 3D, E**), providing a mechanistic explanation for the suppression of CSR observed in stressed mice. Consistent with this, stress significantly reduced CD40 expression on thymic IgD^+^IgM^+^ B cells (**Figure 3F**), further compromising the CD40L–CD40 signaling axis (37).

As professional APCs within the thymus, thymic B cells express high levels of MHC II and efficiently present self-antigens to eliminate autoreactive T cells through apoptosis induction or promotion of regulatory T cell differentiation (38, 39). The antigens presented by B cells comprise both exogenous and endogenous sources, with more than 70% of MHC II–bound peptides in B cells estimated to be derived from intracellular proteins (40). In thymic B cells, endogenous self-antigens may originate from at least three sources. First, Aire was expressed at significantly higher levels in thymic B cells than in peripheral B cells (**Supplementary Figure S1B**), thereby enabling these cells to express a unique repertoire of TRAs (8). Second, CD40L–CD40 and IFN-λ signaling, which drive thymic B cell activation, can reshape the B cell transcriptome and thereby modulate the antigen repertoire available for presentation (9, 18). Third, peptides derived from the variable region of the B cell receptor can be presented as peptide–MHC II complexes on the cell surface (41). Our data demonstrated that stress impairs the antigen-presenting function of thymic B cells, as evidenced by reduced MHC II expression (**Figures 4C–E**) and decreased expression of specific self-antigens (**Figure 4A**). This impairment may be attributed to the significant downregulation of Aire-dependent, CD40L-responsive, and IFN-λ–responsive genes under stress (**Figures 4B**, **3D, E**), which collectively limits the endogenous expression and subsequent presentation of self-antigens.

Notably, although the absolute number of thymic B cells decreased, their proportion among total thymocytes increased (**Figure 2D**), likely owing to the profound loss of CD4^+^CD8^+^ double-positive thymocytes induced by stress (33). Furthermore, stress inhibited the expression of co-stimulatory molecules on IgD^+^IgM^+^ cells, whereas CD40, CD80, CD86, and MHC II expression was significantly upregulated in IgD^-^IgM^-^ cells. This subset-specific response likely reflects intrinsic heterogeneity in the sensitivity of thymic B cells to stress (35). The precise functional consequences of this differential regulation warrant further investigation.

In summary, this study reveals the detrimental impact of stress on thymic B cell homeostasis and function. Specifically, stress suppresses thymic B cell proliferation and impairs key signaling pathways, including CD40L–CD40 and IFN-λ signaling, thereby reducing thymic B cell numbers, impairing the “licensing” process, and compromising antigen-presenting capacity. These findings advance understanding of the mechanisms underlying stress-induced thymic dysfunction and highlight thymic B cells as a previously underappreciated target of stress-mediated immune dysregulation.

## Data Availability

The original contributions presented in the study are publicly available. This data can be found here: http://www.ncbi.nlm.nih.gov/bioproject/1540214, PRJNA1540214.
