# Supplementary material for: Effects of stress on thymic B cells in C57BL/6J mice: phenotypic characterization and mechanistic insights
Source: Front Immunol. 2026 Oct 13;17:1963346. doi: 10.3389/fimmu.2026.1963346 (PMC13710050; doi:10.3389/fimmu.2026.1963346)
Supplement: Supplementary file 1 [file SupplementaryFile1.docx]

Supplementary Material

# 1 Supplementary Figures and Tables

## 1.1 Supplementary Figures

**Supplementary Figure 1.** Landscape of thymic B cells and their response to GCs stimulation revealed by public single-cell datasets. (A–D) Analysis of the single-cell dataset GSE234188; (E–H) Analysis of B cell transcriptomes extracted from the single-cell dataset GSE244229. (A) UMAP projection of CD19⁺ B cells from various tissues. BM, bone marrow; LN, lymph node; SP, spleen; Thy, thymus. (B) Feature plots showing Aire expression across all B cells (left) and within the thymic B cell subset (right); gray indicates undetectable expression. (C) Volcano plot of DEGs between thymic B cells and B cells from other tissues. (D) GO terms enrichment analysis of DEGs (*P_adj_* < 0.05, top 5 most significant terms). BP, Biological Process; CC, Cellular Component; MF, Molecular Function. (E) Volcano plot of DEGs in the 18 h group relative to the control. (F) GO enrichment analysis of DEGs in the 18 h group relative to the control (*P_adj_* < 0.05, top 5 most significant terms). (G, H) GSEA of the 18 h group versus the control using naive B cell gene set (G) or cell cycle gene set (H). Genes were ranked by fold change in descending order.

**Supplementary Figure 2.** Impact of stress on the thymus and DEGs analysis in thymic B cells. Quantitative analysis of thymus weight (A) and total thymocyte count (B) across the three groups (n = 6). (C) H&E staining of the thymus in control and stressed mice (Bar = 50 μm, n = 6). Dashed lines indicate the boundary between the thymic cortex and medulla. (D, E) Volcano plots of DEGs in thymic B cells of the stress 3 d (D) and stress 7 d (E) groups versus the control. DEGs were defined as |log₂FC| ≥ 1 and *P* < 0.05. Transcriptome data were obtained from CD19⁺ B cells sorted from the thymus of mice (n = 3). Red, upregulated; blue, downregulated; gray, non-significant. Data are presented as mean ± SD. Statistical significance was determined by one-way ANOVA followed by Dunnett’s *post hoc* test, or Welch’s ANOVA followed by Dunnett’s T3 *post hoc* test (panel B). ***, *P* < 0.001; ****, *P* < 0.0001.

**Supplementary Figure 3.** Expression of molecules associated with B cell activation assessed by flow cytometry. (A-F) Flow cytometry analysis of CD80 and CD86 expression in thymic B cells from control and stressed mice (n = 6), gated on total CD19⁺ thymic B cells (A, B), IgD⁻IgM⁺ B cells (C, D), and IgD⁻IgM⁻ B cells (E, F) across the three groups (n = 6). (G, H) Flow cytometry analysis of CD40 expression in thymic B cells from control and stressed mice (n = 6), gated on total thymic B cells (G) and IgD^-^IgM^-^ B cells (H). Representative histograms for each group are shown in (A-H). Data are presented as mean ± SD or median (IQR). Statistical significance was determined by one-way ANOVA followed by Dunnett’s *post hoc* test, or Kruskal-Wallis test followed by Dunn’s *post hoc* test (panels E, F, and H). ns, *P* > 0.05; *, *P* < 0.05; **, *P* < 0.01; ***, *P* < 0.001.

**Supplementary Figure 4.** Stress induced transcriptomic alterations in thymic B cells. (A, B) KEGG pathway enrichment analysis of DEGs in the stress 3 d (A) and stress 7 d (B) groups versus the control (n = 3). DEGs were defined as |log₂FC| ≥ 1 and *P* < 0.05. Enriched pathways (*P* < 0.05) were grouped by KEGG second-level categories; the horizontal axis represents the total number of genes within each pathway. (C, D) GO terms enrichment analysis of DEGs in the stress 3 d (C) and stress 7 d (D) groups versus the control (n = 3), showing the top 5 significant terms in the BP, CC, and MF categories (*P* < 0.05).

**Supplementary Figure 5.** Flow cytometric gating strategies for the analysis of thymic B cells. (A) Gating strategy for determining the proportion of total B cells (Protocol 1). Debris and aggregates were sequentially excluded using FSC-A/SSC-A and FSC-A/FSC-H plots. Live singlets were identified by excluding 7-AAD-positive events, and total B cells were subsequently gated as CD19⁺ events. (B) Gating strategy for analyzing the proportions of B cell subsets and their surface marker expression (CD40, CD80, CD86, and MHC II) (Protocol 2). CD4⁺ and CD8α⁺ T cells were depleted via negative magnetic selection prior to staining to enrich the B cell population. The initial gating steps were identical to those in (A). Within the B cell gate, B cell subsets were defined according to IgD and IgM expression. The surface marker expression levels were then quantified by MFI.

**Supplementary Figure 6.** Positive mouse tissue control for CD19 IHC staining. Representative IHC images of mouse spleen tissue showing CD19 expression in the white pulp (Bar = 50 μm).

## 1.2 Supplementary Tables

**Supplementary Table 1.** Information of antibodies used in flow cytometry/IHC.

| Antibody | Conjugate | Clone | Company | Catalogue | Host species | Application |
| --- | --- | --- | --- | --- | --- | --- |
| CD19 | FITC | 1D3 | Proteintech | 65290 | Rat | 1:200 |
| CD19 | \ |  | Proteintech | 27949-1-AP | Rabbit | 1:500 |
| CD19 | PE | MB19-1 | Thermo | 12-0191-82 | Mouse | 1:200 |
| CD4 | Biotin | RM4-5 | BD Biosciences | 553045 | Rat | 1:100 |
| CD8α | Biotin | 53-6.7 | BD Biosciences | 553028 | Rat | 1:200 |
| CD40 | BV786 | 3/23 | BD Biosciences | 740891 | Rat | 1:100 |
| IgD | BV510 | 11-26c.2a | BD Biosciences | 563110 | Rat | 1:100 |
| IgM | PE-Cy7 | R6-60.2 | BD Biosciences | 552867 | Rat | 1:100 |
| CD80 | APC | 16-10A1 | BD Biosciences | 560016 | Hamster | 1:100 |
| CD86 | PE | GL1 | BD Biosciences | 553692 | Rat | 1:100 |
| I-A/I-E | RY703 | 2G9 | BD Biosciences | 771455 | Rat | 1:100 |
| CD16/32 | \ | 2.4G2 | Starter Biotechnology | S0B0599-100T | Rat | 1:50 |
| Anti-Rabbit IgG | HRP | \ | Proteintech | RGAR001 | Goat | 1:1000 |

**Supplementary Table 2.** Enriched GO terms for downregulated DEGs in the GSE234188 dataset. Of the 77 downregulated DEGs, 71 were annotated in the GO background. Terms with *P_adj_* <0.05 were considered significantly enriched. A total of 38 biological process (BP) terms and 2 cellular component (CC) terms were enriched; the top 10 most significant BP terms and the 2 CC terms are presented.

| Ontology | Description | GeneRatio | BgRatio | RichFactor | FoldEnrichment | zScore | *P* | *P_adj_* |
| --- | --- | --- | --- | --- | --- | --- | --- | --- |
| BP | T cell migration | 4/71 | 69/28905 | 0.057971 | 23.60073 | 9.326857 | 2.56E-05 | 0.035391 |
|  | positive regulation of neutrophil chemotaxis | 3/71 | 37/28905 | 0.081081 | 33.00914 | 9.667678 | 0.000104 | 0.035391 |
|  | positive regulation of granulocyte chemotaxis | 3/71 | 40/28905 | 0.075 | 30.53345 | 9.275004 | 0.000131 | 0.035391 |
|  | aerobic respiration | 5/71 | 199/28905 | 0.025126 | 10.22896 | 6.482593 | 0.000132 | 0.035391 |
|  | lymphocyte migration | 4/71 | 112/28905 | 0.035714 | 14.53974 | 7.124125 | 0.00017 | 0.035391 |
|  | regulation of neutrophil chemotaxis | 3/71 | 45/28905 | 0.066667 | 27.14085 | 8.708301 | 0.000187 | 0.035391 |
|  | contractile actin filament bundle assembly | 4/71 | 117/28905 | 0.034188 | 13.91838 | 6.947859 | 0.000201 | 0.035391 |
|  | stress fiber assembly | 4/71 | 117/28905 | 0.034188 | 13.91838 | 6.947859 | 0.000201 | 0.035391 |
|  | actomyosin structure organization | 5/71 | 223/28905 | 0.022422 | 9.128087 | 6.046329 | 0.000225 | 0.035391 |
|  | regulation of vascular permeability | 3/71 | 49/28905 | 0.061224 | 24.92527 | 8.317493 | 0.000241 | 0.035391 |
| CC | sex chromosome | 3/71 | 42/28618 | 0.071429 | 28.79074 | 8.988456 | 0.000157 | 0.026798 |
|  | GBAF complex | 2/71 | 14/28618 | 0.142857 | 57.58149 | 10.56054 | 0.000542 | 0.046315 |
